# Classification of Sesame Oil Based on Processing-Originated Differences in the Volatile Organic Compound Profile by a Colorimetric Sensor

**DOI:** 10.3390/foods13203230

**Published:** 2024-10-11

**Authors:** Tianyi Liu, Hai-Ming Shi, Untzizu Elejalde, Xiaodong Chen

**Affiliations:** 1 Wilmar Innovation Center, Wilmar International HQ, 28 Biopolis Rd., Singapore 138568, Singapore; tianyi.liu@sg.wilmar-intl.com; 2 School of Materials Science and Engineering, Nanyang Technological University, Block N4.1, 50 Nanyang Ave., Singapore 639798, Singapore; 3 Wilmar (Shanghai) Biotechnology Research & Development Center Co., Ltd., 118 Gao Dong Rd., Shanghai 200137, China; shihaiming@cn.wilmar-intl.com

**Keywords:** colorimetric sensor, VOC, sesame oil, refined oil, hot pressing, small milling

## Abstract

Fragrant edible sesame oil is popular for its unique aroma. The aroma of sesame oil is determined by its volatile organic compound (VOC) profile. Sesame oils produced by different techniques could have different VOC profiles. In addition, blending fragrant sesame oil with refined oil could also alter the VOC profile of the final product. Current practices in aroma analysis, such as sensory evaluation and gas chromatography (GC), still face many restraints. Hence, there is a need for alternatives. We present a novel 14-unit multiplexed paper-based colorimetric sensor for fragrant sesame oil VOC analysis. The sensor was designed to visualize the VOC profile as a color “fingerprint”. The sensor was validated with 55 branded sesame oil samples produced by two different techniques, i.e., hot pressing and small milling; the experimental results suggested a processing dependency in color VOC fingerprints. The sensor also demonstrated the potential to detect the change in sesame oil VOC profile due to blending with refined oil, with an estimated limit of detection down to 20% *v*/*v* of the refined oil. The colorimetric sensor might be used as a simple, rapid, and cost-effective analytical tool in the production and quality control of fragrant sesame oil.

## 1. Introduction

Aroma is an important quality indicator of edible plant oil. Aroma is determined by the volatile organic compounds (VOCs) present in oil, which vary depending on factors like raw materials, processing techniques, cooking practices, and freshness [1,2,3,4]. Sesame oil is a popular type of fragrant edible plant oil with a consumption history dating back to the Babylonian era [5]. Making up approximately 50% of the total mass, oil can be easily obtained from sesame seeds by various oil pressing techniques, including cold pressing, hot pressing, and small milling (“Xiao Mo” in Chinese), as well as solvent extraction [6,7]. In both hot pressing and small milling, mechanical force is used to extract oil from roasted sesame seeds. However, in small milling, hot water is used to separate oil and undesirable components by their difference in density [8]. Hence, small milling is sometimes referred to as the hot-water floatation method [9]. In both processes, the oil produced does not undergo further refining, so as to preserve the aroma. Existing studies have found that fragrant sesame oil produced by different techniques has different VOC profiles [10]. Fragrant sesame oil is sometimes blended with refined oil for various purposes [11,12,13]. Since refining removes most of the VOCs from oil, the resultant blended oil has a VOC profile that is different from pure fragrant sesame oil [14]. Such differences in VOC profiles affect the sensory attributes and, subsequently, consumer preference [9,10,15]. Considering the increasing demand for high-quality fragrant sesame oil, aroma analysis is always a topic of interest in the industry.

In the food industry, the aroma of sesame oil is commonly assessed by human sensory evaluators. Despite being closer to consumer perception, sensory analysis is constrained by many factors, including a long training time and the subjective nature of individuals’ feelings [16,17,18]. Over the years, various laboratory-based methods have been adopted to assist humans in aroma analysis, with gas chromatography (GC) being the most widely adopted method. However, discrepancies in signal interpretation exist between GC-based techniques and human olfaction. Unlike human olfaction, where the electrochemical signals generated by olfactory receptors are integrated into one scent “fingerprint”, GC-based techniques are analytical in nature and hence, information on aroma is fragmentized into molecular compositions [19]. To mimic humans in odor sensing, artificial noses utilizing a diverse range of materials and mechanisms have been developed since 1982 [20]. Similar to the human olfactory system, an artificial nose consists of a sensor array with cross-reactiveness and a pattern recognition system that functions similarly to the human brain [21,22].

Although research on analyzing edible plant oil VOCs with artificial noses has been ongoing for the past two decades, most of the studies employed general-purpose commercial electronic noses (E-noses), making it difficult to correlate the results to the chemistry of oil VOCs [23]. Moreover, E-noses require sophisticated and expensive equipment for data collection and a professional background for data interpretation. Hence, there is a need to explore a cost-effective and simple alternative. Colorimetry is a principle frequently used in VOC sensors, whereby the presence of target VOCs is reflected as a color change. Compared with other sensing techniques, colorimetric sensors could achieve higher sample throughput and faster response without compromising sensitivity and accuracy. Most importantly, sensor readouts can be observed by the naked eye, and no complex data processing is required. As a result, colorimetric sensors have become a topic of interest in food testing, especially in the freshness monitoring of perishable food products like meat and fruits [24,25,26,27]. Although some prior work on the colorimetric sensing of edible oil VOCs has been performed, earlier research mainly focused on VOCs released by degraded oil using a sensor made with a single colorimetric indicator. Xie et al. fabricated a Schiff’s reagent-loaded poly(vinyl alcohol) nanofiber mat to detect aldehydic VOCs released during oil oxidation [28]. Alternatively, Liu et al. reported a hydroxylamine sulfate pH indicator-impregnated chitin film for the same purpose [29]. Recently, colorimetric VOC sensor arrays have been introduced to detect adulteration in edible oil. Huang et al. used a nine-unit colorimetric VOC sensor comprising pH indicators and metalloporphyrins to detect soybean oil and sesame oil adulteration in extra virgin olive oil [30]. Mehdizadeh et al. reported a six-unit pH indicator-based VOC sensor to detect sunflower oil and sesame oil adulteration in quince oil [31]. Both studies reported achieving satisfying results. However, little is known about the applicability of colorimetric sensors in analyzing the processing-originated differences in the aroma of fragrant sesame oil or the detection of refined oil in fragrant sesame oil.

**Figure 1:**
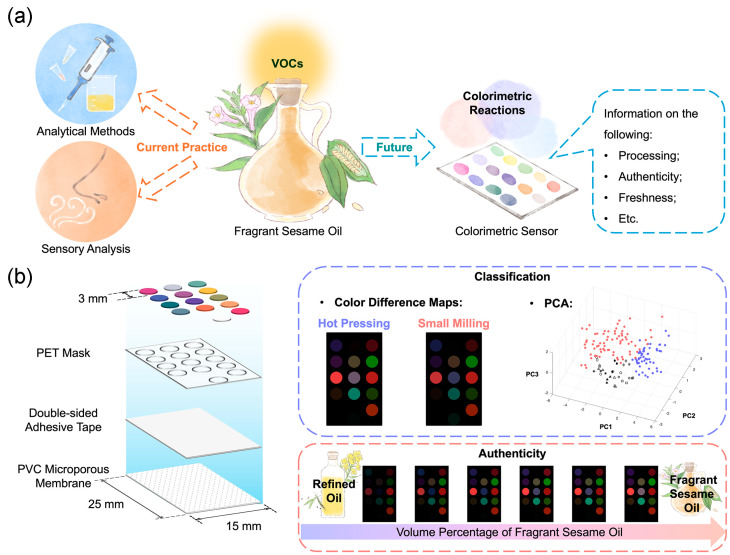
(**a**) A colorimetric sensor array was proposed as a rapid, simple, and low-cost alternative to the current practice for fragrant sesame oil VOC analysis; information on sesame oil properties can be easily derived from the colorimetric VOC “fingerprint” generated by the sensor. (**b**) The sensor comprised 14 paper-based sensing units, each acting as a colorimetric receptor. The sensor demonstrated the potential to classify fragrant sesame oil samples produced by hot pressing and small milling based on their VOCs and to detect refined oil in fragrant sesame oil. On the principal component analysis (PCA) plot: red dots—small-milled sesame oil samples; blue-dots—hot-pressed sesame oil samples; grayscale symbols—suspected adulterated hot-pressed sesame oil samples.

Here, we present a 14-unit paper-based colorimetric sensor for sesame oil VOC analysis, in which each sensing unit could act as a colorimetric “receptor” (Figure 1a). We report the first sensor multiplexing study that combines three groups of colorimetric indicators into one sensor array and targets key VOCs in sesame oil. pH indicators, carbonyl-sensitive indicators, and solvatochromic dyes were used to build this sensor array. The sensor was validated with both chemical standards and 55 commercial sesame oil samples. The results generated by the sensor were compared to gas chromatography–mass spectrometry (GC-MS) data and analyzed by both cluster and regression analyses. Experimentally, the sensor demonstrated the potential to be a simple and rapid tool for the visualization of the VOC profile of fragrant sesame oil. On one hand, it could reveal the difference in aroma caused by the processing technique; on the other hand, it could also detect the change in the VOC profile due to the presence of refined oil in fragrant sesame oil (Figure 1b).

## 2. Materials and Methods

### 2.1. Reagents

Bromocresol Green sodium salt, Bromocresol Purple sodium salt (dye content 90%), Bromothymol Blue (analytical grade), Chrome Azurol S (dye content 50%), Congo Red, Methyl Red (analytical grade), Pararosaniline base (dye content 95%), Reichardt’s Dye (dye content 90%), Resazurin sodium salt, Xylenol Orange tetrasodium salt, Schiff’s reagent, potassium hydroxide (KOH), sodium carbonate (Na_2_CO_3_), hydroxylamine sulfate, acetic acid, hexanoic acid, pentanal, heptanal, benzaldehyde, phenylacetaldehyde, furfural, ethanol, acetone, methanol (HPLC-grade), and n-alkanes (C4–C30) were obtained from Sigma-Aldrich (Sigma-Aldrich, St. Louis, MO, USA). Brooker’s Merocyanine was obtained from Shanghai Kingmorn Biotechnology (Shanghai Kingmorn Biotechnology Co., Shanghai, China). 2-Heptanol was obtained from Adamas Reagent (Adamas Reagent Co., Ltd., Shanghai, China). Sesamin, sesamolin, asarinin, and sesamol were purchased from Sinopharm Medicine Holding (Sinopharm Medicine Holding Co., Ltd., Shanghai, China). Litmus indicator solution (1.0% ± 0.05%) was obtained from Hebei Hongzhuangyuan Education & Technology (Hebei Hongzhuangyuan Education & Technology Ltd., Shijiazhuang, China).

### 2.2. Sensor Fabrication

**Figure 2:**
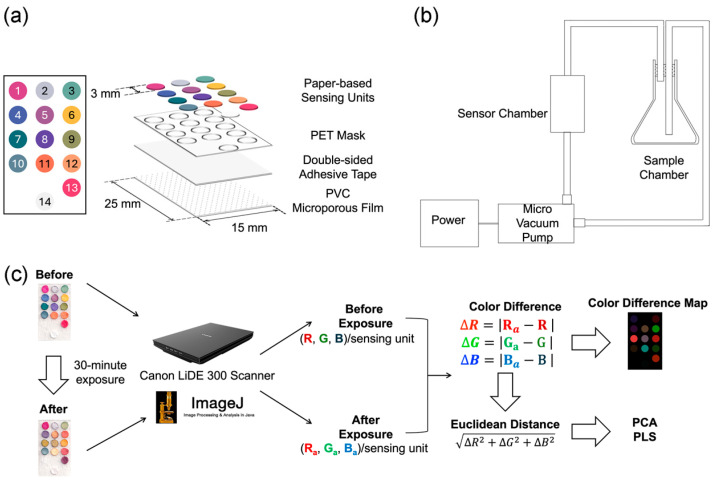
(**a**) The architecture and dimensions of the 14-unit colorimetric sensor. The chemical compositions of the receptors can be found in Supplementary Information S1. (**b**) A schematic illustration of the sensing device. The sensing device consisted of a sample chamber, a sensor chamber, and a micro vacuum pump powered by a portable power source. (**c**) A flowchart of the sensing procedures. The images of the sensor were acquired with a desktop scanner; digital color information was extracted using ImageJ 1.53a (Wayne Rasband, National Institutes of Health, Bethesda, MD, USA) and subjected to further analysis.

In total, 14 indicator solutions were used to fabricate the colorimetric receptors. Indicator solution Nos. 2–5 contained pH indicators; solution Nos. 6–9 contained both pH indicators and basic chemicals (KOH/ Na_2_CO_3_). Indicator solution Nos. 1, 13, and 14 contained carbonyl-sensitive dyes. Indicator solution Nos. 10–12 contained solvatochromic dyes that could change color upon inter-reaction with polar VOCs. All indicator solutions were prepared as described in Supplementary Information S1. To fabricate the colorimetric receptors, Grade 1 qualitative filter paper (Advantec Toyo Kaisha, Ltd., Tokyo, Japan) was cut into circular pieces (∅ = 3 mm) using a hole puncher; each colorimetric receptor was made by dipping the circular piece into the indicator solution for 1 s, followed by drying. For receptor Nos. 2–5 and 13, drying was performed in the laboratory oven at 60 °C for 30 min; for receptor Nos. 6–9, drying was performed in the laboratory oven at 60 °C for 15 min; for receptor Nos. 1, 10–12, drying was performed at room temperature for 30 min; and for receptor No. 14, drying was performed at room temperature overnight. The dried receptors were sealed in zip-lock bags and stored in the dark for further use. All receptors were prepared one day prior to sensing. The sensor was assembled by attaching the 14 receptors to a 1.5 cm × 2.5 cm polyvinyl chloride (PVC) microporous film using double-sided adhesive tape. To position the receptors, a mask with the sensor array pattern was cut out from polyethylene terephthalate (PET) film using a Silhouette Cameo desktop cutting machine (Silhouette America, Inc., Lindon, UT, USA) (Figure 2a).

### 2.3. Sensor Validation with Chemical Standards

To demonstrate if the colorimetric sensor could react with common VOCs in food, acetic acid, hexanoic acid, pentanal, heptanal, benzaldehyde, phenylacetaldehyde, and furfural were used as VOC standards. The validation experiment was performed by exposing the sensor to vapors of the chemical standards [27]. Briefly, 2 μL of a liquid chemical was dropped onto the bottom of a 5 L chamber; the liquid chemical was allowed to evaporate for 30 min. A scanned image of the sensor before exposure to the chemical vapor was obtained using a Canon LiDE 300 scanner (Canon Inc., Tokyo, Japan). The colorimetric sensor was then placed inside the chamber to react with the vapor. After 30 min, the sensor was taken out and the scanned image was acquired.

### 2.4. Edible Oil Samples

The properties of edible oil produced by large companies tend to be different from those produced by small manufacturers because of differences in the scale of machinery, the processing system, and regulatory compliance [32,33,34]. To better understand the sensor’s applicability in actual practice, a total of 55 branded commercial sesame oil samples were collected from China and used in this study, including 29 hot-pressed (HP) sesame oil samples and 26 small-milled (XM) sesame oil samples. Compared with unbranded oil, branded oil products tend to have better and more consistent quality [35,36]. The 55 commercial sesame oil samples were from 21 brands that were labeled with letters (A to U). One hot-pressed sample (labeled as “HP-S”) and one small-milled sample (labeled as “XM-S”) from the same manufacturer were used as the standards of authentic sesame oil. To prepare blended sesame oil, one hot-pressed fragrant sesame oil sample and one refined oil (refined canola oil) sample were purchased from supermarkets in Singapore. Hot-pressed sesame oil is commonly used in blended sesame oil as it has a relatively lower production cost and stronger flavor. The refined oil and sesame oil were mixed in 4 different volume ratios (refined oil/fragrant sesame oil = 8:2/5:5/2:8/1:9), each to a final volume of 50 mL. Therefore, together with the two pure samples, a total of 6 samples were tested in the experiment.

### 2.5. Headspace Solid-Phase Microextraction–Gas Chromatography (HS-SPME-GC)

The two authentic sesame oil samples and 37 randomly selected commercial samples (19 hot-pressed samples and 18 small-milled samples) were analyzed by headspace solid phase microextraction–gas chromatography (HS-SPME-GC). The experiment was performed at Wilmar (Shanghai) Biotechnology Research & Development Center. 2-Heptanol was used as the internal standard for semi-quantitative analysis. N-alkanes (C4–C30) were used to calculate the linear retention index (LRI). For VOC analysis, 5 g of sesame oil and 20 μL of the 2-heptanol solution (1000 μg/mL) were placed in a 20 mL vial. The vial was tightly capped with polytetrafluoroethylene (PTFE)/silicone septum. Equilibration was performed at 80 °C for 20 min. The VOCs were extracted at 50 °C for 50 min using a 50/30 μm divinylbenzene–carboxen–polydimethylsiloxane (DVB/CAR/PDMS) StableFlex fiber (Supelco Inc., Bellefonte, PA, USA) from the headspace of the vial (the vial was under orbital shaking at 450 rpm). The fiber was immediately desorbed for 2 min in a GC injection port operating in splitless mode at 250 °C. The fiber was conditioned at 300 °C under helium flow for 1 h in accordance with the manufacturer’s recommendations prior to use. Periodic fiber blanks were run to ensure the absence of contaminants and/or carryover.

The separation of VOCs was performed with an Agilent 8890 GC system coupled with a 5977B Mass Selective Detector (MSD) (Agilent Technologies Inc., Santa Clara, CA, USA), using an FFAP column (60 m ×0.25 mm, 0.25 μm, Agilent Technologies Inc., Paolo Alto, CA, USA). Helium was used as the carrier gas at the flow rate of 1 mL/min. The column temperature was initially held at 40 °C for 1.5 min, increased to 100 °C at the rate of 10 °C/min, and held for 5 min; the temperature was then increased to 150 °C at the rate of 2 °C/min, held for 8 min, increased to 185 °C at the rate of 10 °C/min, and then increased to 240 °C at the rate of 20 °C/min and held for 16 min. The GC-MS interface temperature was 250 °C, and the ion source temperature was 230 °C. Electron impact mass spectra were generated at 70 eV and acquired in full-scan mode with the range of 35–350 *m*/*z*. Data were collected and processed using the NIST 17 Mass Spectral Library. For the preliminary identification of the compounds, a matching factor score greater than 800 was accepted. Further confirmation of the tentative identification results was achieved by qualitatively comparing the retention indices with those of the standard compounds.

### 2.6. Colorimetric Sensing of VOCs from Fragrant Sesame Oil

The colorimetric sensing of VOCs from fragrant sesame oil samples collected in Section 2.4 was performed with a customized device, which consisted of a sample chamber, a sensor chamber, and an AM370 micro vacuum pump (AIM Technology, Dongguan, China) connected by flexible polymer tubing and adaptors (Figure 2b). The micro vacuum pump was powered by a portable power bank. For each test, 5 mL of oil was used, and three sensors were tested in parallel. For the experiment on the detection of refined oil, the sensing of each sample was repeated 5 times by analyzing 5 aliquots (5 mL/aliquot); three sensors were tested in parallel for each aliquot. All experiments were performed at room temperature with a sensing duration of 30 min. Images of the sensor, before and after exposure to the sample VOCs, were obtained using a Canon LiDE 300 scanner (Canon Inc., Tokyo, Japan).

### 2.7. Data Analysis

Digital color information was extracted using ImageJ 1.53a. The color difference was calculated as the absolute difference between the R, G, and B (red, green, and blue) values before and after exposure:

∆*R* = |*R*_After_ − *R*_Before_|

∆*G* = |*G*_After_ − *G*_Before_|

∆*B* = |*B*_After_ − *B*_Before_|

A color difference map was constructed by using the ∆*R*, ∆*G*, and ∆*B* values. Each receptor on the color difference map thus had a color (*R*′, *G*′, *B*′), whereby *R*′ = ∆*R*, *G*′ = ∆*G*, *B*′ = ∆*B*. The Euclidean distance between the colors of each receptor, before and after exposure was calculated. The data acquisition and processing procedures are summarized in Figure 2c.

The calculated Euclidean distances were subjected to principal component analysis (PCA). PCA is a classic multivariate statistical analysis technique that reduces the dimension of data. Principal components (PCs) are linear combinations of the original variables that enable the data to explain the maximum variance. By plotting the first two or three PCs, closely related data can be identified as a cluster. For the experiment on the detection of refined oil, data were analyzed by partial least squares (PLS) regression analysis in addition to PCA. PLS regression analysis is commonly used to find the linear regression model between two sets of data, the predictors and the responses, by projecting them into a new space. In this case, the Euclidean distance was used as the predictor, and the concentration of refined oil (in % *v*/*v*) was the response. Both PCA and PLSR were performed with the built-in functions of MATLAB_R2023b software (MathWorks, Inc., Natick, MA, USA).

### 2.8. Reversed-Phase Liquid Chromatography (RP-LC)

The lignan contents of the selected hot-pressed sesame oil samples were analyzed by reversed-phase liquid chromatography (RP-LC). Sesamin, sesamolin, asarinin, and sesamol were used as the lignan standards (purity >97%). The lignan stock solutions (200 μg/mL in methanol) were prepared individually. The working standard solutions were prepared by diluting the stock solutions. All solutions were stored at −20 °C in the dark. Calibration curves were constructed using the working standard solutions prior to the analysis of sesame oil samples.

The lignan concentration was determined by the external standard method. For RP-LC analysis, 0.5 g of sesame oil was weighed and dissolved in 8 mL of methanol. The mixture was first vortexed for 2 min, centrifuged at 6000 rpm for 3 min, and then filtered through a 0.22 μ nylon syringe filter (ANPEL Scientific Instrument Co., Ltd., Shanghai, China). For each sample, 20 μL of the filtered solution was used for analysis. RP-LC was performed with an Agilent 1200 HPLC system coupled with a UV/Vis detector (Agilent Technologies Inc., Santa Clara, CA, USA). A Venusil XBP C18 column (5 μm, 250 × 4.6 mm) was used. The mobile phase was methanol/water (in a volume ratio of 8:2), at the flow rate of 1 mL/min. The column temperature was 30 °C. The absorption wavelength was 287 nm.

## 3. Results

### 3.1. Sensor Validation with Chemical Standards

**Figure 3:**
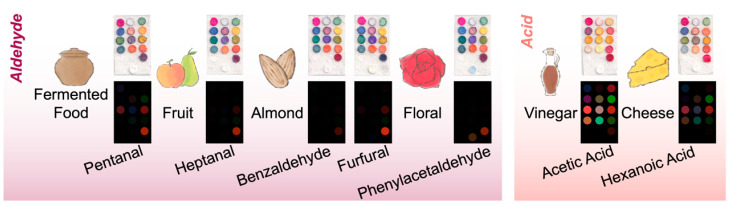
The scanned images and corresponding color difference maps generated by the sensor after exposure to common VOCs in food (for visualization, the color range of 0–167 was expanded to the 8-bit color range). The sensory attributes of the VOCs are included. The 14 receptors demonstrated different affinity to various VOCs, indicating the cross-reactive nature of the sensor.

In this study, the sensor’s response to seven common VOCs found in food products was investigated. These VOCs have different sensory attributes, which could be pleasant or unpleasant depending on the type of food in which they are found (Figure 3). The sensor demonstrated cross-reactiveness, as each receptor could react with multiple VOCs.

In general, receptor Nos. 1–12 were more sensitive to acids, while receptor Nos. 13 and 14 were more sensitive to aldehydes. Receptor Nos. 13 and 14 were formulated with chemicals that could react specifically with aldehydes. Receptor No. 13 was based on the reaction between aldehydes and hydroxylamine sulfate; the acid produced by this reaction could be easily detected using a suitable pH indicator [29]. Notice that receptor No. 13 could undergo a slight color change in response to acids, as a trace amount of Na_2_CO_3_ was added to adjust the color of the indicator solution. Yet such color change was insignificant compared to that caused by aldehydes. Receptor No. 14 was made with Schiff’s reagent, which is commonly used to detect aldehydes colorimetrically. Interestingly, the receptor was found to turn pale yellow and light blue upon reaction with furfural and phenylacetaldehyde vapors, respectively, instead of the commonly observed pink color caused by other aldehydes. Among the acid-sensitive receptors, receptor Nos. 1, 6–9, 11, and 12 were also found to undergo color change upon exposure to certain aldehydes, especially No. 9, which could change color in response to pentanal, heptanal, and furfural. This could be attributed to the chemical properties of the indicators. Receptor No. 1 was loaded with Pararosaniline base, which has aryl amine groups that can react with carbonyl groups [37,38]. Receptor Nos. 6–9 contained pH indicators mixed with Na_2_CO_3_ or KOH. The color change could be due to the reaction between aldehydes and bases [27]. In addition, receptor Nos. 11 and 12 were solvatochromic dyes that were introduced for non-specific detection of polar VOCs. Since the receptors possessed different affinity to the VOCs, the results suggested that the color fingerprint might be used to estimate the types of VOCs present in a given sample.

### 3.2. Sensor Validation with Sesame Oil Standards

**Figure 4:**
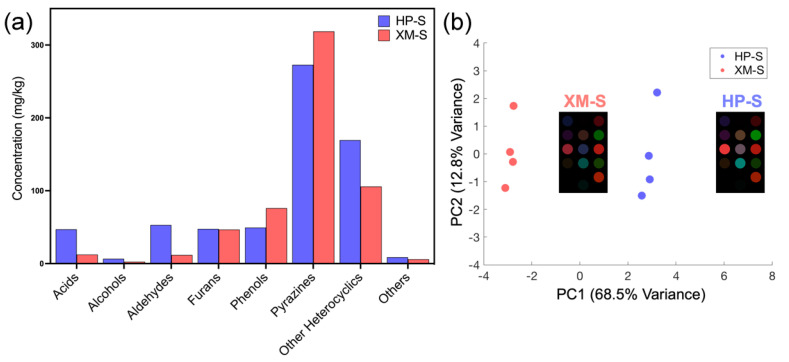
(**a**) Concentrations of major VOCs in the two authentic sesame oil samples measured using HS-SPME-GC analysis. The same groups of VOCs were detected in both HP-S and XM-S, yet the exact composition showed slight variation. (**b**) The PCA result based on the Euclidean distance values obtained by the colorimetric sensor array in response to the VOCs of HP-S and XM-S. HP-S and XM-S were clearly separated by PC1. Insets: average color difference maps of the two standards; HP-S produced stronger signals than XM-S, especially in receptor Nos. 5, 7, 8, and 11.

Analysis of the results by HS-SPME-GC as described in Section 2.5 resulted in the identification of eight groups of VOCs in both HP-S and XM-S, with pyrazine being the most abundant group (Figure 4a). Pyrazines are chemicals responsible for the unique aroma of roasted food [39]. However, considering its abundance in both types of fragrant sesame oil, the slight difference in pyrazine concentration could thus be less significant in discriminating the processing technique. However, it was noticed that HP-S had a much higher content of acids and aldehydes, which are VOCs that could be easily detected by our sensor, as shown in Section 3.1. Using the colorimetric sensor array, it was found that HP-S and XM-S produced color fingerprints of similar patterns, yet the sensor detected much stronger signals from HP-S, especially by receptor Nos. 5, 7, 8, and 11 (Figure 4b, insets). The Euclidean distances calculated from the difference in R, G, and B values were used for cluster analysis. PCA was performed on the three replicates of each sample as well as the numerical averages. The first two principal components accounted for over 80% of the total variance. It can be seen that the clusters of HP-S and XM-S were clearly separated by the first principal component (PC1) (Figure 4b).

### 3.3. Commercial Fragrant Sesame Oil

#### 3.3.1. GC-MS Analysis

**Figure 5:**
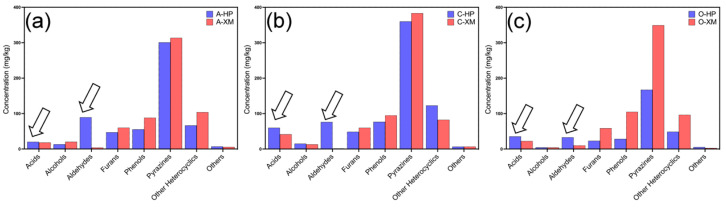
The VOC profiles of the hot-pressed and small-milled samples from (**a**) Brand A, (**b**) Brand C, and (**c**) Brand O. For products from the same brand, the hot-pressed sample always contained more volatile acids and aldehydes (indicated by the arrows).

The mean total VOC concentrations in commercial hot-pressed samples and small-milled samples were 551.92 mg/kg and 522.47 mg/kg, respectively (Table S2-1). The major types of VOCs identified were the same as those in Section 3.2. The trend in HP-S and XM-S was also applicable to commercial samples, as hot-pressed samples generally had a significantly higher content of acids and aldehydes than small-milled samples. A close examination of the types of acidic and aldehydic VOCs identified in the two types of fragrant sesame oil revealed that hot-pressed fragrant sesame oil tended to contain more volatile acids and aldehydes with low molecular weights (Table S2-2). Acetic acid, which was found in most of the hot-pressed samples, was not detected in any of the 18 small-milled samples. Likewise, alkyl aldehydes with five to nine carbon atoms, such as 3-methyl-butanal, pentanal, and heptanal, showed more frequent occurrence in hot-pressed samples. This trend could also be observed in hot-pressed and small-milled sesame oil products from the same brand, as the hot-pressed sesame oil product always contained more acids and aldehydes than the small-milled counterpart, regardless of the total VOC concentration (Figure 5).

#### 3.3.2. Colorimetric Sensing

**Figure 6:**
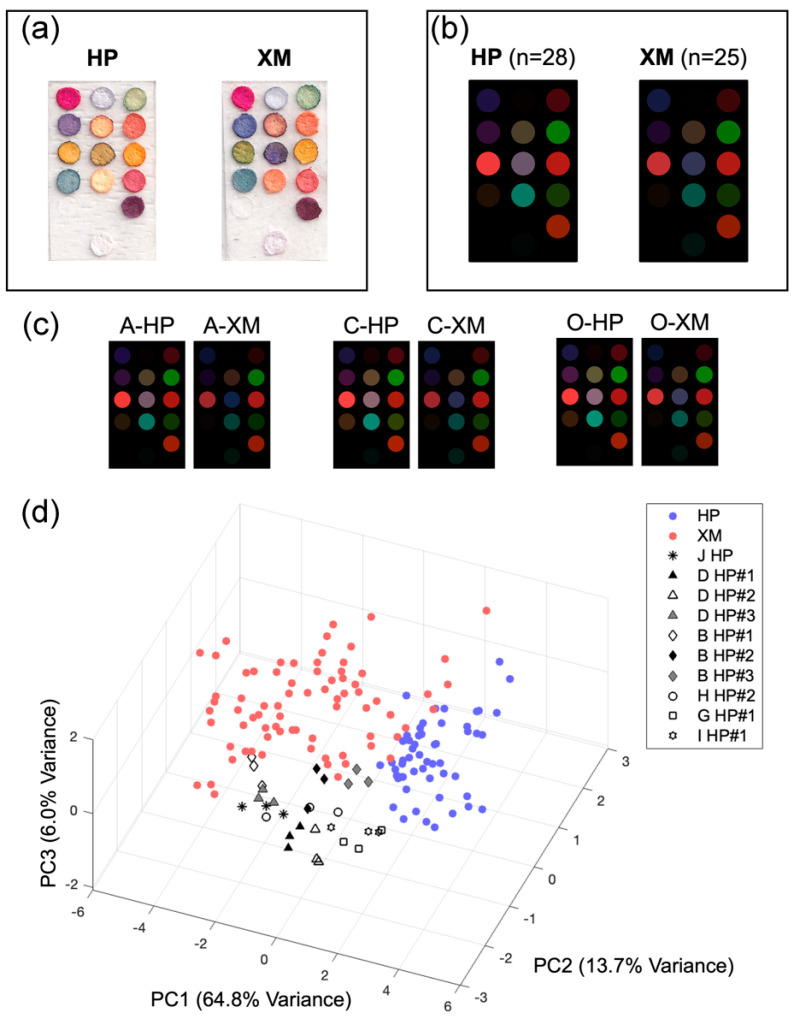
(**a**) Typical scanned images of the colorimetric sensor array after exposure to hot-pressed (HP) and small-milled (XM) samples. Differences in the color fingerprints could be easily observed the naked eye. (**b**) The average color difference maps of commercial hot-pressed and small-milled samples. Compared with small-milled samples, hot-pressed samples generally produced more significant color changes, especially in sensing unit Nos. 5, 7, 8, and 11. (**c**) The color difference maps of hot-pressed and small-milled samples from Brands A, C, and O. For products from the same brand, the hot-pressed sample always produced stronger signals. (**d**) PCA results of 53 commercial samples; hot-pressed samples with normal color difference maps, hot-pressed samples with abnormal color difference maps, and small-milled samples are marked by blue, grayscale, and red symbols, respectively. Most hot-pressed samples clustered in the region of positive PC1 values, while most small-milled samples were found in the region of negative PC1 values. Among hot-pressed samples, some were found to produce less significant color changes; these samples were separated from the other hot-pressed samples by PC2 values.

Typical scanned images and average color difference maps of the commercial hot-pressed and small-milled samples are shown in Figure 6a and Figure 6b, respectively. Most of the receptors could be activated after 30 min of exposure. Similar to sesame oil standards, hot-pressed samples generally produced stronger signals than small-milled samples, especially in receptor Nos. 5, 7, 8, and 11. This trend could also be observed in hot-pressed and small-milled sesame oil products from the same brand (Figure 6c). According to the color fingerprints of the chemical standards in Section 3.1, these four receptors were sensitive to small-molecule acids and aldehydes, especially acetic acid. The results obtained by the colorimetric sensor thus suggested a higher content of acids and aldehydes in hot-pressed sesame oil, which also agreed with GC-MS analysis. Interestingly, small-milled samples were found to induce greater color change in receptor No. 14 (indicated by a dark-teal dot on the color difference map). Greater differences were observed in receptor Nos. 3–8, 10, 11, and 14, which consisted of pH-sensitive, aldehyde-sensitive, and solvatochromic indicators (Figure S2). Data collected by these receptors was used for PCA. The total variance explained by the first three principal components was 84.5% (Figure 6d). The clustering behavior of fragrant sesame oil samples suggested a dependency on processing technique, as hot-pressed samples and small-milled samples formed two distinct clusters. Similar to the finding in Section 3.2, hot-pressed and small-milled samples were largely differentiated by PC1.

Among the 28 hot-pressed samples, 10 samples were found to have color difference maps with weaker signals compared with the rest, and these samples were separated from the majority by PC2. Among the abnormal hot-pressed samples, the sample from Brand I was a blended sesame oil product (containing both refined and fragrant sesame oil). Hence, the remaining abnormal hot-pressed samples were also suspected of containing refined oil.

**Figure 7:**
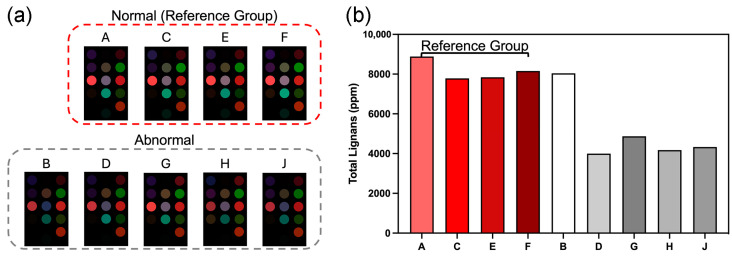
(**a**) The color difference maps of selected normal hot-pressed samples (from Brands A, C, E, and F) and abnormal hot-pressed samples (from Brands B, D, G, H, and J). Stronger signals were detected from normal hot-pressed samples, especially by receptor Nos. 7, 8, and 11. (**b**) Total lignan contents of the selected commercial hot-pressed samples. Samples with abnormal color difference maps generally had lower lignan contents, except Brand B. A low lignan content suggested that refined oil might be present.

The refining process could affect the lignan content in sesame oil. Research has found that refined sesame oil tends to have lower lignan content [7]. The lignan contents of the abnormal hot-pressed samples were thus analyzed by reversed-phase liquid chromatography (RP-LC) as described in Section 2.8 (Figure 7). Brands D, G, H, and J were found to have significantly lower lignan contents compared with the reference group, which suggested that the samples from these brands might contain refined oil. However, Brand B had a lignan content comparable to those with normal color difference maps. Thus, Brand B might contain either a low amount of refined oil or might be produced by technique(s) other than hot pressing and small milling.

### 3.4. Colorimetric Detection of Refined Oil in Blended Sesame Oil

**Figure 8:**
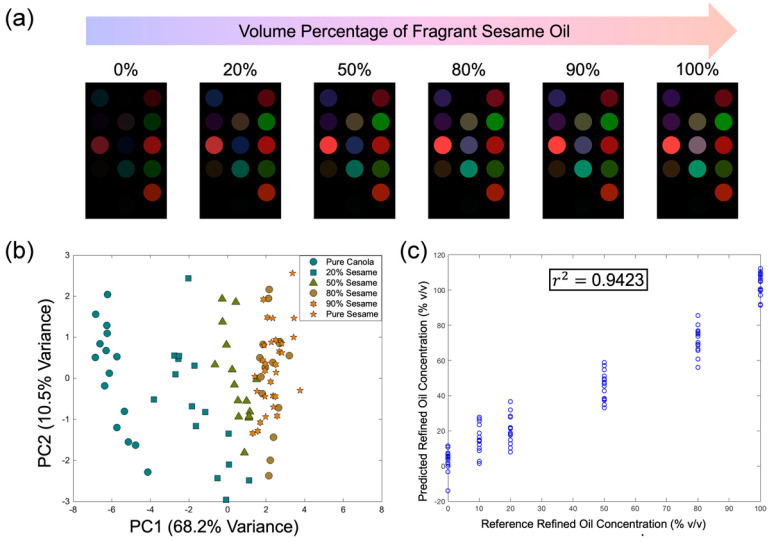
(**a**) The average color difference maps of samples containing various volume percentages of fragrant sesame oil. As the volume percentage of fragrant sesame oil increased, the signals detected by the sensor became stronger; the color difference map of blended sesame oil containing more than 80% *v*/*v* of fragrant sesame oil closely resembled that of the pure sesame oil. (**b**) PCA result. Refined oil samples formed a cluster that was clearly separated from that of pure sesame oil; as the volume percentage of refined oil decreased, the PC1 value became more positive; when the percentage decreased to 20% *v*/*v*, the clusters started to overlap. (**c**) PLS regression analysis result. With 9 PLS components selected, an r^2^ value of 0.9423 could be obtained, suggesting a reasonably strong correlation.

The results obtained with commercial sesame oil samples in Section 3.3 suggested that our sensor might be able to detect refined oil in fragrant sesame oil. To evaluate the sensing performance systematically, a series of blended fragrant sesame oils was prepared. Refined canola oil was used as a sample of common refined oil because of its negligible aroma. The average color difference maps of blended samples are shown in Figure 8a. Pure refined oil, which has low aroma intensity, could only produce weak signals; as the volume percentage of sesame oil increased, the color intensity increased gradually, and the color fingerprint resembled more of the color fingerprint of typical hot-pressed fragrant sesame oil.

PCA was performed using the Euclidean distances of all 14 sensing units. The first two principal components accounted for 78.7% of the total variance (Figure 8b). Overlap among clusters could be observed when the volume percentage of refined oil dropped below 20%. In addition to PCA, PLS regression analysis was used to analyze the results. Here, the Euclidean distances were used as the predictors, while the volume percentages of refined oil were the responses. The PLS regression model was first constructed by selecting an appropriate number of PLS components (i.e., latent variables). It was found that when nine components were selected, r^2^ = 0.9423, suggesting a reasonably strong correlation, and there was no significant increase in the r^2^ value when the number of PLS components increased further. Hence, the number of PLS components was set to nine for subsequent analysis (Figure 8c). The limit of detection (LOD) of the regression model was estimated by the three-sigma method [40]. Briefly, replicates of a blank were analyzed by the analytical method, which was the PLS regression model in this case, and the responses were converted into concentration units, the standard deviation (SD) was calculated, and the LOD was estimated as LOD = 3 SD. Using the predicted refined oil concentration at 0% *v*/*v* of refined oil, the standard deviation was calculated as SD = 6.491% *v*/*v*, and the LOD was thus estimated to be 19.5% *v*/*v* of refined oil. It is worth noting that the LOD of the PLS model would vary slightly if the VOC profiles of the sesame oil and refined oil changed. To further demonstrate the reproducibility, the experiment was repeated with sesame oil of different brands and batches (Supplementary Information S3), and in all cases, PLS regression models with r^2^ > 0.9 could be obtained.

## 4. Discussion

In this study, a 14-unit paper-based colorimetric sensor was used to analyze the VOCs in fragrant sesame oil by sensing the headspace gas while the sample was placed in a sealed sample chamber. The headspace gas in the sample chamber was analyzed as VOCs only constitute a small fraction of the total mass, while over 90% of the liquid phase is made up of triglycerides [41]. Moreover, fragrant sesame oil generally has a dark color, as brown pigments are formed by the Maillard reaction during the roasting process, and the oil obtained is not subjected to any further refining process; hence, the pigments will remain in the final product, giving fragrant sesame oil its dark color. All these factors make it less feasible to perform VOC analysis directly on liquid oil. However, simply exposing the sensor to the headspace gas would require a longer time for colorimetric reactions to proceed, and a long sensing duration was reported in most of the existing studies [28,29,31]. Heating is sometimes used to shorten the sensing time [30]. In this study, the VOC analysis duration of the colorimetric sensor was shortened without the need for heating, and naked-eye observable color changes could be achieved in 30 min at room temperature. To achieve this, a micro vacuum pump was incorporated into the sensing system to facilitate gas circulation, and a cellulose-based material was selected as the substrate to load indicator dyes, instead of other commonly used hydrophobic polymer membranes. Filter paper, as an easily available cellulose-based material, is hydrophilic. Water plays an important role in many chemical reactions, such as proton transfer between immobilized dye molecules and gaseous analytes, which is necessary for pH indicators to change color. The water molecules adsorbed could thus facilitate various reactions to occur [42,43,44]. Moreover, filter paper is intrinsically porous and readily available, making it ideal for disposable gas-sensing devices.

In this study, both GC-MS analysis and colorimetric sensing confirmed the differences in the content of acidic and aldehydic VOCs between hot-pressed sesame oil and small-milled sesame oil. It was found that hot-pressed sesame oil tended to contain more acids and aldehydes. Such a difference could be caused by the unique aqueous extraction step in the small milling process. Volatile acids and aldehydes tend to have higher water solubility, and hence, they can be easily removed from the oil phase by aqueous extraction [15]. Many volatile acids and aldehydes have been identified as off-flavor compounds in edible plant oil [45,46]. Hence, the presence of such VOCs affects the aroma of fragrant sesame oil. In a study conducted by Dong et al., the sensory evaluation suggested that small milling could produce sesame oil with the most preferable aroma among various techniques [10]. Chen et al. compared small-milled and hot-pressed sesame oil samples, and concluded that small-milled sesame oil had stronger and better aroma intensity than the roasted oil [9]. Such difference in aroma could be attributed to the lower content of volatile acids and aldehydes in small-milled sesame oil [15]. Since volatile acids and aldehydes are odorants that could affect the aroma of sesame oil, they should be given more attention in the production and quality control of fragrant sesame oil. Our colorimetric sensor was highly sensitive to such VOCs, the difference between hot-pressed sesame oil and small-milled sesame oil could be amplified. Principal component analysis (PCA) suggested that despite slight brand-to-brand variation, the processing technique (i.e., hot pressing or small milling) had the dominant effect on the color VOC fingerprint of the final oil product. In addition, the colorimetric sensor array could effectively combine the information on sample VOCs into a color fingerprint; the color changes exhibited by the receptors were clearly observable by the naked eye, and minimal data processing was thus required. These factors make the sensor a promising rapid analytical tool for quality control in the sesame oil production line.

In addition to classification by the processing technique, the sensor demonstrated the potential to detect the presence of refined oil in hot-pressed sesame oil. The deviation in the color VOC fingerprint of blended sesame oil from that of pure fragrant sesame oil was closely related to the concentration of refined oil. It could thus directly monitor the dilution of aroma during the formulation and production of blended sesame oil. In addition, VOC analysis has been proposed as an alternative method to examine the authenticity of edible oil; the VOC profile could provide more useful information on authenticity when the components in a blended oil have similar triglyceride/fatty acid profiles [47]. Moreover, when refined oil is illegally added to sesame oil, the final product is considered adulterated. Since the sensor could be operated without any strict requirement on environmental conditions, it might also be used to perform on-site inspection during market surveillance, saving time and effort in sampling and transferring to a centralized laboratory.

However, the sensor demonstrated in this study was developed for the purpose of rapid classification and authentication of fragrant sesame oil. Future research can thus aim at establishing the correlation between the color VOC fingerprints generated by the sensor and the concentrations of target VOCs. Moreover, since the naked eye-observable color changes exhibited by the colorimetric receptors can be easily captured by a camera, advanced image processing algorithms, such as deep convolutional neural networks (DCNNs), can be used to further enhance the user-friendliness of the sensing device.

## 5. Conclusions

In this study, a 14-unit colorimetric sensor array combining three groups of colorimetric indicators was developed to analyze the VOCs of fragrant sesame oil. The sensor was simple to fabricate and highly portable; the analysis result was presented as a color VOC fingerprint from which information could be easily inferred. Such properties could give the sensor an advantage in actual practice. An extensive study on 55 branded sesame oil samples suggested that the sensor could differentiate hot-pressed sesame oil from small-milled sesame oil based on the processing-originated differences in VOC profile, in particular, the content of volatile acids and aldehydes. The sensor also demonstrated the potential to detect the presence of refined oil in blended sesame oil. Most importantly, whereas most studies until now have demonstrated the detection of fragrant oil quality deterioration, in this work, for the first time, we show that the classification of fragrant oils based on the differences emanating from processing techniques is possible. We believe that the concept and methodology demonstrated in this study can also be applied to develop colorimetric sensors for other applications in the food industry, such as the classification and authentication of common condiments and post-harvest monitoring of fruits. These colorimetric sensors can be used as simple, rapid, and cost-effective analytical tools to assist food quality control in the future.

## 6. Patents

The authors have a patent pending to Nanyang Technological University (Singapore) and Wilmar International Ltd. (Singapore).

## Data Availability

The original contributions presented in the study are included in the article/Supplementary Material, further inquiries can be directed to the corresponding authors.

## Supplementary Materials

The following supporting information can be downloaded at https://www.mdpi.com/article/10.3390/foods13203230/s1: Table S1: Compositions of the 14 indicator solutions; Figure S1: Sensor architecture, dimensions, and colorimetric receptor arrangement; Table S2-1: Concentrations of major VOCs identified in commercial sesame oil samples; Table S2-2: Concentrations of acids and aldehydes identified in commercial sesame oil samples; Figure S2: Euclidean distances of all 14 receptors; Figure S3-1: The average color difference maps of samples containing various volume percentages of sesame oil; Figure S3-2: The PCA and PLSR analysis results of adulteration experiments with (a) sesame oil sample 1, (b) sesame oil sample 2, and (c) sesame oil sample 3.
