# Supplementary material for: Classification of Sesame Oil Based on Processing-Originated Differences in the Volatile Organic Compound Profile by a Colorimetric Sensor
Source: Foods. 2024 Oct 11;13(20):3230. doi: 10.3390/foods13203230 (PMC11507991; doi:10.3390/foods13203230)
Supplement: Supplementary file 1 [file foods-13-03230-s001.zip › foods-3189210-supplementary.pdf]

Classification of Sesame Oil Based on Processing-Originated Differences in the Volatile Organic Compound Profile by a Colorimetric Sensor

Supplementary Information

S1 Fabrication of Colorimetric Sensor

The sensor comprised 14 sensing units made with 14 different indicator solutions (Table S1). Based on their chemical properties, the indicators could be classified into three categories: pH indicators, carbonyl-sensitive indicators, and solvatochromic dyes. Indicators No. 2 - 9 were pH-sensitive. Among the pH-sensitive indicators, No. 6 - 9 were formulated with basic chemicals, i.e. sodium carbonate (Na_2CO_3) or potassium hydroxide (KOH). Addition of basic chemicals could enhance the sensitivity towards acidic VOCs. Indicators No. 1, 13, and 14 were carbonyl-sensitive indicators; indicator No. 1 was also sensitive to acids, while No. 13 and No. 14 targeted aldehydes. Indicators No. 10 - 12 were solvatochromic dyes for detection of polar volatile organic compounds (VOCs).

Table S1 Compositions of the 14 indicator solutions.

No.	Indicator	Solvent	Concentration
1	Pararosaniline base	Ethanol	2 mg/mL
2	Litmus indicator solution	As received.	
3	Bromocresol Green	20% Ethanol	1 mg/mL
4	Resazurin sodium salt	Ethanol	2 mg/mL
5	Xylenol Orange tetrasodium salt	DI water	2 mg/mL
6	Methyl Red + Na_2CO_3	Ethanol-DI water	Methyl Red: 2 mg/mL<br>Na_2CO_3: 0.5 mg/mL

No.	Indicator	Solvent	Concentration
7	Bromothymol Blue (BTB) + Na_2CO_3	Ethanol-DI water	BTB: 2 mg/mL<br>Na_2CO_3: 0.5 mg/mL
8	Bromocresol Purple (BCP) + KOH	Ethanol-DI water	BCP: 2 mg/mL<br>KOH: 0.5 mg/mL
9	BTB + KOH	Ethanol-DI water	BTB: 2 mg/mL<br>KOH: 0.25 mg/mL
10	Reichardt's Dye	Ethanol (10% v/v)-acetone (90% v/v)	5 mg/mL
11	Brooker's Merocyanine	Ethanol	2 mg/mL
12	Chrome Azurol S	Ethanol	2 mg/mL
13	Hydroxylamine sulfate + Congo Red	DI water	10 mg/mL<br>With 0.5 mg/mL Congo Red
14	Schiff's reagent	As received.	

Each indicator solution was prepared by dissolving appropriate amount of solid indicator dye in a suitable solvent to the concentration stated in Table S1. For indicator No. 6 – 9, stock solutions of indicator dyes (5 mg/mL in ethanol) and bases (20 mg/mL in DI water) were first prepared; the indicator solutions used to fabricate the receptors were then prepared by mixing the stock solutions in appropriate ratio; ethanol was

added so that the final concentrations of the indicator dye and the base reached the concentrations stated in Table S1. For indicator No. 13, trace amount of aqueous Na_2CO_3 was added to adjust the color (pH) of the indicator solution. Indicator solutions No. 2 and No. 14 were used as received.

The sensor architecture, dimensions, and colorimetric receptor arrangement are shown in Figure S1. The number on each receptor represents the corresponding indicator solution in Table S1.

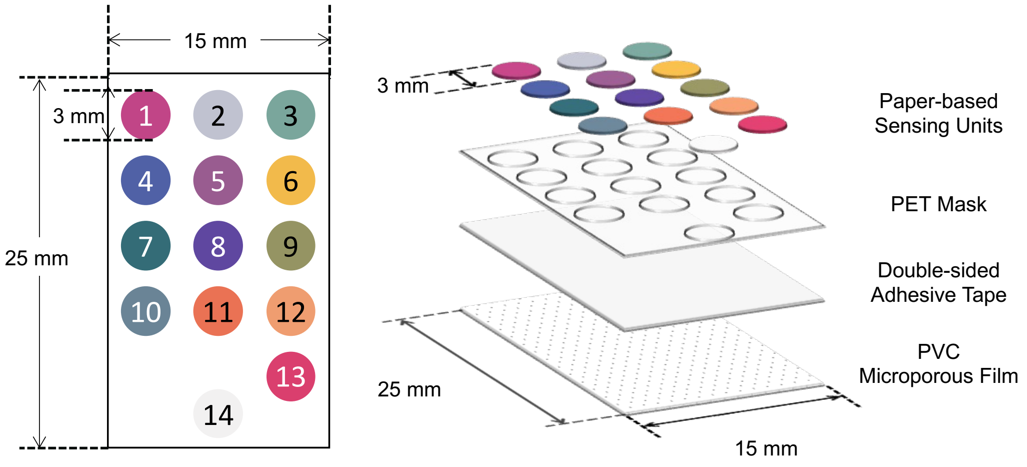

Figure S1 Sensor architecture, dimensions, and colorimetric receptor arrangement.

S2 Analysis Results of Commercial Sesame Oil Samples

The overall VOC profiles of the selected commercial sesame oil samples (19 hot-pressed samples and 18 small-milled samples) are shown in Table S2-1. The average concentrations of volatile acids and aldehydes detected in these samples are summarized in Table S2-2.

Table S2-1 Concentrations of major VOCs identified in commercial sesame oil samples.

Compounds	Concentration (mg/kg)			
	Hot-pressed (n=19)		Small-milled (n=18)	
	Mean	SD	Mean	SD
Acids	47.12	21.99	17.48	8.80
Alcohols	7.51	4.23	9.95	6.68
Aldehydes	66.19	35.47	12.52	10.83
Furans	37.23	12.68	42.15	14.45
Phenols	48.87	19.71	61.28	26.38
Pyrazines	265.94	88.22	298.68	68.18
Other Heterocyclics	71.75	46.56	73.10	26.86
Others	7.31	2.98	7.32	3.95
Total	551.92	164.76	522.47	131.92

Table S2-2 Concentrations of acids and aldehydes identified in commercial sesame oil samples.

Compounds	Concentration (mg/kg)			
	Hot-pressed		Small-milled	
	(n=19)		(n=18)	
	Mean	SD	Mean	SD
Acetic acid	10.52	12.58	-	-
Propanoic acid	1.19	1.25	0.12	0.51
2-Butenoic acid, 3-methyl-	-	-	0.04	0.17
Pentanoic acid	0.45	0.93	0.47	1.13
Hexanoic acid	14.55	5.82	6.96	2.90
Heptanoic acid	4.21	1.56	1.03	1.11
Nonanoic acid	11.78	9.28	4.33	3.65
Octanoic acid	4.12	6.45	3.95	2.95
n-Decanoic acid	0.31	1.02	0.58	2.11
Total acids	47.12	21.99	17.48	8.80
Butanal, 3-methyl-	1.44	2.73	0.27	0.63
Pentanal	0.70	1.79	-	-
Hexanal	16.53	12.44	1.17	2.11
Heptanal	1.12	2.69	-	-
Octanal	1.60	4.08	-	-
Nonanal	1.27	5.53	-	-

2-Octenal, (E)-	8.24	4.10	1.74	2.41
2,4-Nonadienal	1.09	1.13	-	-
2-Nonenal	-	-	0.11	0.33
2,4-Decadienal	16.51	9.07	2.79	4.80
2-Decenal, (E)-	6.41	5.06	-	-
Decanal	0.06	0.27	-	-
2-Undecenal	5.94	4.33	-	-
Benzaldehyde	2.39	1.34	2.54	2.95
Piperonal	1.59	3.34	0.92	1.31
Benzaldehyde, 4-methyl-	0.38	1.16	1.69	2.09
Benzeneacetaldehyde, .alpha.-ethylidene-	0.92	1.01	1.28	0.79
Total aldehydes	66.19	35.47	12.52	10.83
*Furfural	4.75	2.11	4.99	2.22

*Furfural is a furan with aldehyde functional group. Furfural was found to be able to react with certain indicators in this study. However, no significant difference was found in furfural concentration between the studied hot-pressed and small-milled samples.

The color difference was quantified by Euclidean distance (Figure S2). Greater difference in Euclidean distance could be observed in sensing units No. 3 - 8, 10, 11 and 14, i.e. the difference in mean Euclidean distance was greater than 5 arbitrary units. Hot-pressed samples generally had larger Euclidean distance, except in sensing unit No. 14.

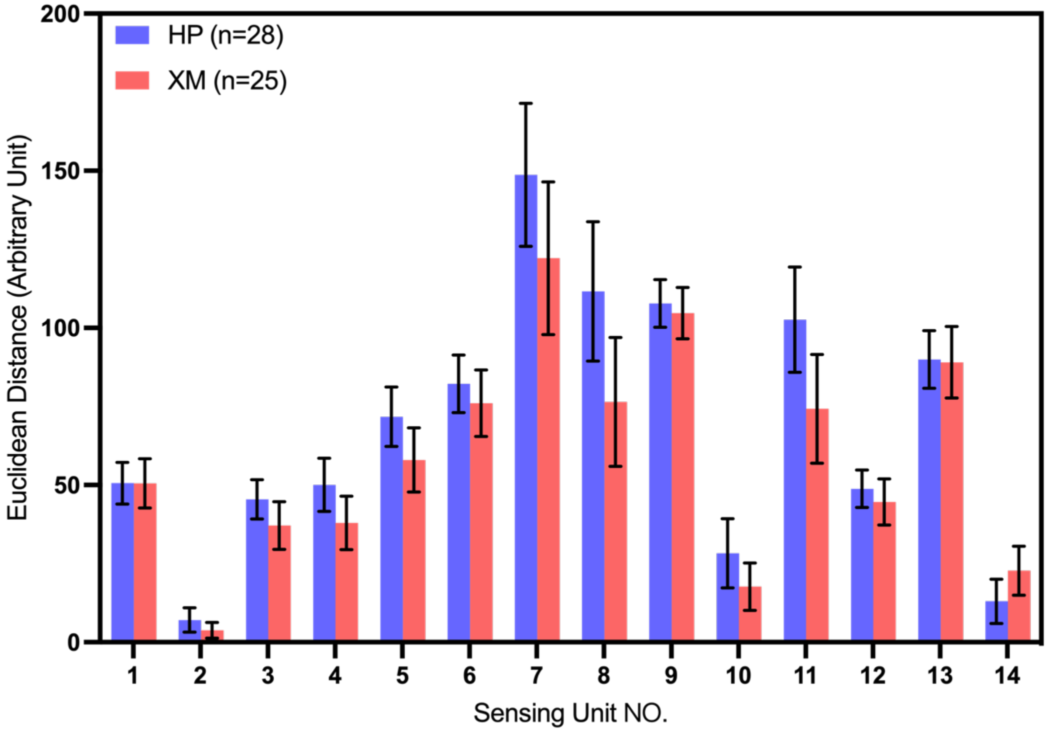

Figure S2 Euclidean distances of all 14 receptors. The total number of samples tested was 53. Greater differences could be observed in receptors No. 3 - 8, 10, 11 and 14. Hot-pressed samples generally had larger Euclidean distances, except in receptor No. 14. HP: hot-pressed; XM: small-milled.

S3 Blending with Refined Oil

The color difference maps of pure refined oil, blended sesame oil, and pure fragrant sesame oil are shown in Figure S3-1. The color intensity increased gradually with the percentage of fragrant sesame oil.

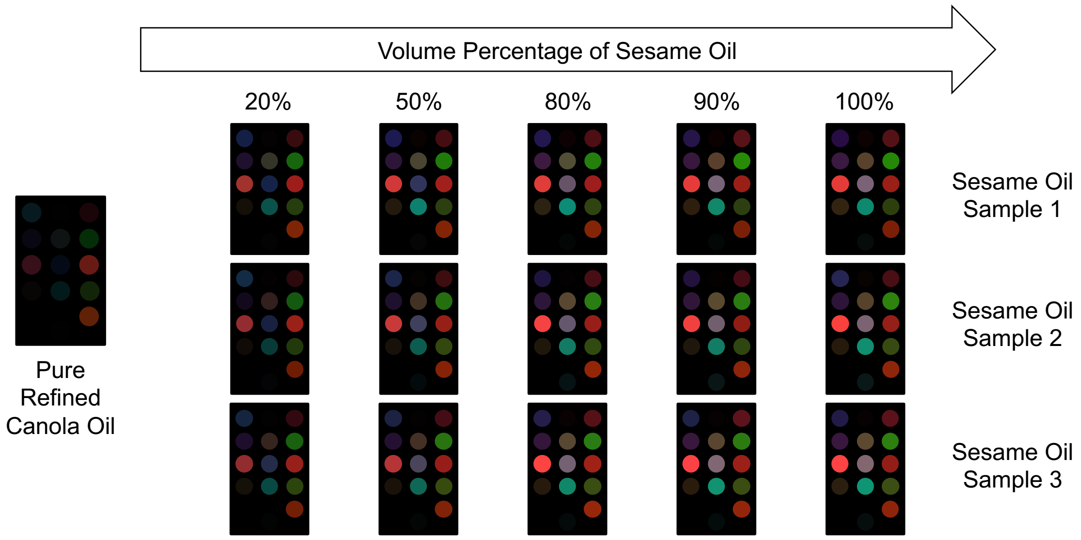

Figure S3-1 The average color difference maps of samples containing various volume percentages of sesame oil.

The PCA and PLSR analysis results of the three sets of experiments are shown in Figure S3-2. Clustering analysis by PCA revealed common trends in all three experiments: as the percentage of refined oil decreased, the separation between clusters decreased, especially for those containing less than 20% v/v of refined oil. For PLSR analysis, 5 PLS components (i.e. latent variables) were used to construct the PLS model. The resultant r^2 values exceeded 0.9 in all three experiments, indicating that the observations could be well explained by the corresponding regression models.

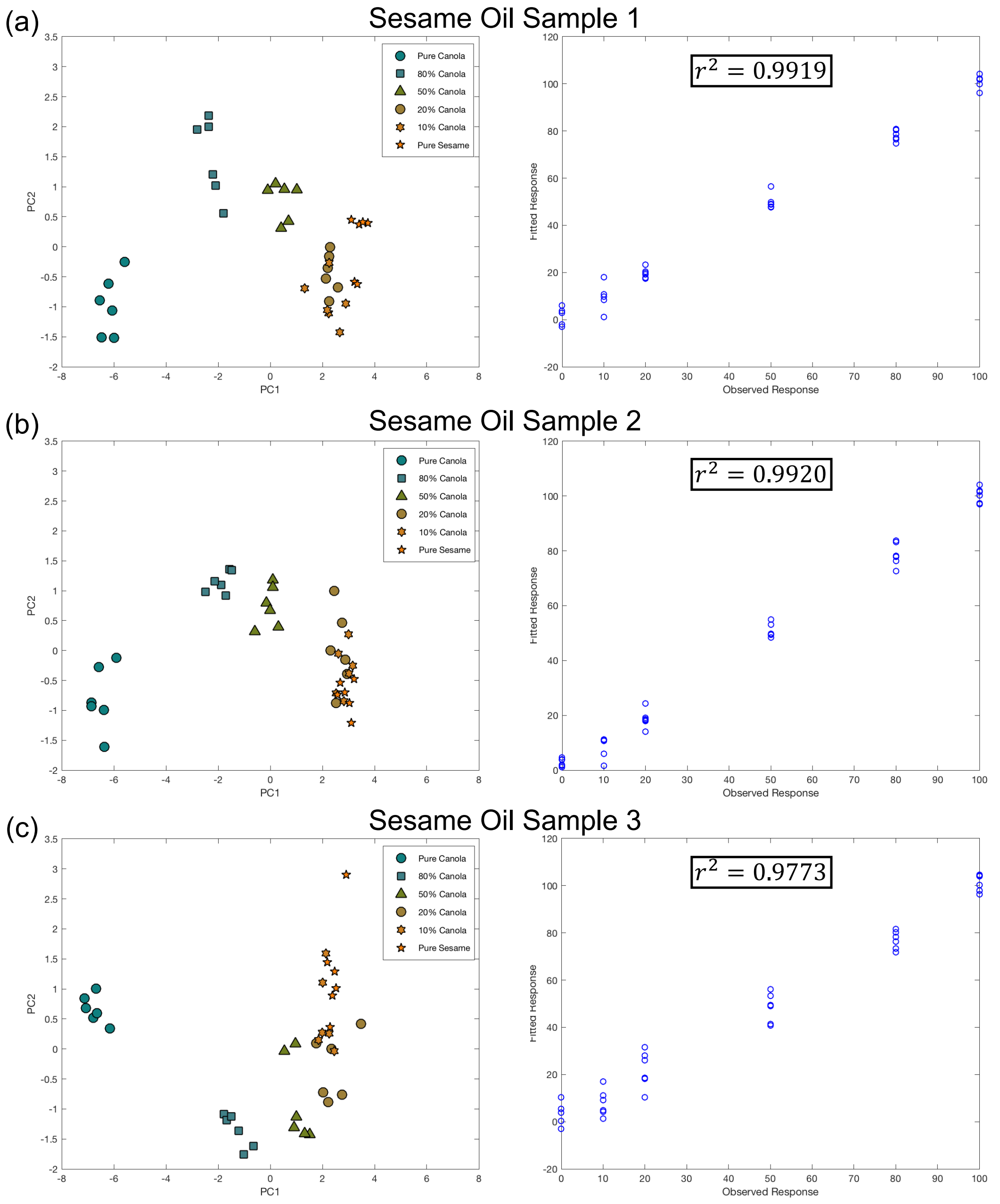

Figure S3-2 The PCA and PLSR analysis results of adulteration experiments with: (a) sesame oil sample 1, (b) sesame oil sample 2, and (c) sesame oil sample 3. Observed response: reference refined oil concentration; fitted response: predicted refined oil concentration.
